# Associations between COVID-19 Vaccination and Behavioural Intention to Receive Seasonal Influenza Vaccination among Chinese Older Adults: A Population-Based Random Telephone Survey

**DOI:** 10.3390/vaccines11071213

**Published:** 2023-07-06

**Authors:** Xue Liang, Jiming Li, Yuan Fang, Qingpeng Zhang, Martin C. S. Wong, Fuk-yuen Yu, Danhua Ye, Paul Shing-fong Chan, Joseph Kawuki, Siyu Chen, Phoenix K. H. Mo, Zixin Wang

**Affiliations:** 1 Jockey Club School of Public Health and Primary Care, Faculty of Medicine, The Chinese University of Hong Kong, Hong Kong, China; 1155187741@link.cuhk.edu.hk (X.L.); 1155180448@link.cuhk.edu.hk (J.L.); wong_martin@cuhk.edu.hk (M.C.S.W.); benfyyu@cuhk.edu.hk (F.-y.Y.); danhuaye@cuhk.edu.hk (D.Y.); pchan@link.cuhk.edu.hk (P.S.-f.C.); joseks256@gmail.com (J.K.); chensiyu@link.cuhk.edu.hk (S.C.); phoenix.mo@cuhk.edu.hk (P.K.H.M.); 2 Department of Health and Physical Education, The Education University of Hong Kong, Hong Kong, China; lunajoef@gmail.com; 3 Musketeers Foundation Institute of Data Science, The University of Hong Kong, Hong Kong, China; qpzhang@hku.hk; 4 Department of Pharmacology and Pharmacy, LKS Faculty of Medicine, The University of Hong Kong, Hong Kong, China

**Keywords:** seasonal influenza, vaccination, COVID-19 perceptions, older adults, Hong Kong

## Abstract

During the Coronavirus Disease 2019 (COVID-19) pandemic, seasonal influenza remained a significant health threat for older adults. Seasonal influenza vaccination (SIV) is highly effective and safe for older adults. This study investigated the associations of COVID-19 vaccination, perceptions related to COVID-19 and SIV, with the behavioural intention to receive SIV among older adults in Hong Kong, China. A random telephone survey was conducted among 440 community-dwelling Hong Kong residents aged 65 years or above, between November 2021 and January 2022. Among the participants, 55.7% intended to receive SIV in the next year. After adjustment for significant background characteristics, concern about whether SIV and COVID-19 vaccination would negatively affect each other was associated with a lower intention to receive SIV, while a perceived higher risk of co-infection with COVID-19 and seasonal influenza was positively associated with the dependent variable. In addition, the perceived severe consequences of seasonal influenza, perceived benefits of SIV, received cues to action from doctors and participants’ family members or friends, and the perception that more older people would receive SIV was associated with a higher behavioural intention. Future programmes promoting SIV among older adults should modify perceptions related to COVID-19 vaccination and SIV at the same time.

## 1. Introduction

Globally, the disease burden caused by seasonal influenza remains heavy [1]. In the 2022/2023 season, the Center for Disease Control and Prevention (CDC) estimated that there were 27–54 million flu illnesses and 19000–58000 flu deaths in the United States [2]. In China, there were over 1.14 million seasonal influenza cases reported in 2020 [3]. Seasonal influenza mainly affects older adults (i.e., individuals aged 65 years or above) [4]. In Hong Kong, China, where this study was conducted, individuals aged ≥ 65 years have the highest hospitalisation rate caused by seasonal influenza [5].

Seasonal influenza vaccination (SIV) is highly effective in preventing seasonal influenza and all-cause mortality among individuals aged ≥ 65 years [6,7]. The World Health Organization (WHO) recommends annual SIV for adults aged ≥ 65 years [1]. The WHO suggests that all the currently available SIVs are effective and should be considered for older adults [8]. However, in Hong Kong, the SIV coverage among individuals aged ≥ 65 years was only 44.7% in the 2020–2021 season and 40.4% in the 2021–2022 season [9]. Such a coverage level was lower than that in England (70.5%) and the United States (65.3%) [10]. Moreover, as SIV is routinely offered to older residents of residential care homes and older inpatients in public hospitals in Hong Kong, the coverage of SIV was even lower among community-dwelling older adults [11].

During the Coronavirus Disease 2019 (COVID-19) pandemic, seasonal influenza remained a significant health threat. Studies showed that people with a co-infection of seasonal influenza and COVID-19 had much higher risk of mortality than those with neither influenza nor COVID-19 or with COVID-19 only [12,13,14]. It is suggested that SIV could help reduce the risk and severity of COVID-19 [15,16,17]. In addition, SIV has been considered as a protective factor against cardiovascular morbidity and mortality in patients with COVID-19 [18]. Therefore, promoting SIV uptake among older adults during the pandemic is important.

Across countries, numerous studies have investigated factors influencing SIV uptake among older adults [19], including seven studies conducted in Hong Kong [20,21,22,23,24,25,26]. Some similar facilitators and barriers of SIV uptake among older adults were reported by these studies, including socio-demographic, lifestyle, health status, medical service utilization and knowledge and perceptions of seasonal influenza [19]. However, these studies were predominantly performed before the COVID-19 pandemic. Levels of hesitancy and influencing factors of SIV uptake might be different in the post-COVID-19 era.

As compared to the 2019–2020 season, a higher SIV uptake was recorded in the 2020–2021 season among hospital-based workers in Italy [27], the African-American pregnant population in the United States [28], and individuals aged above 50 years in Canada [29]. However, a slight decrease in SIV uptake was found among older adults in Hong Kong [9]. Very few studies have investigated how COVID-19 and the rollout of the COVID-19 vaccination would affect acceptance of SIV. Considering oneself at high risk of COVID-19, and having more concerns of COVID-19 were facilitators to receiving SIV among participants in the United Kingdom [30] and Canada [29], while the decreasing number of COVID-19 vaccination doses was negatively associated with SIV uptake in Israel [31]. Co-infection with COVID-19 and seasonal influenza is not rare [32,33]. It is possible that older adults who perceive a higher chance of co-infection would have a stronger motivation to receive SIV. At the time when this study was conducted, a 14-day interval between the SIV and COVID-19 vaccination was recommended by the Hong Kong government [34]. Older adults may have concerns about whether these two types of vaccinations would have a negative impact on each other. Such concerns may become a barrier to receive SIV and/or COVID-19 vaccination. However, no studies have tested such a hypothesis.

To address the knowledge gap, we therefore investigated behavioural intention to receive SIV during the COVID-19 pandemic among community-dwelling older adults in Hong Kong, China. Potential associated factors considered by this study included background characteristics; uptake of COVID-19 vaccination; perceptions related to COVID-19 and COVID-19 vaccination; and knowledge, perceptions and peer influences about SIV.

## 2. Materials and Methods

### 2.1. Study Design

**Figure 1:**
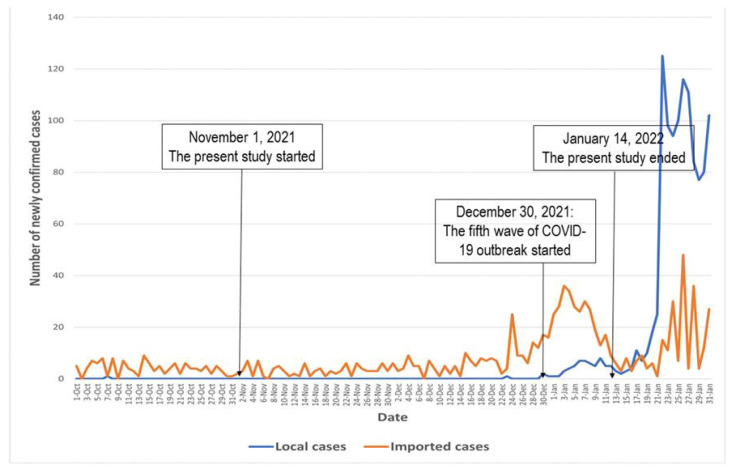
The new cases of COVID-19 during the study period in Hong Kong.

Between 1 November 2021 and 14 January 2022, a telephone-based random survey among community-dwelling older adults was conducted in Hong Kong [35]. The new cases of COVID-19 during the study period in Hong Kong are presented in Figure 1. The COVID-19 outbreak did not have a significant impact on SIV services in Hong Kong. No suspension or interruption of SIV services was reported during the study period.

### 2.2. Participants and Sample Size Planning

The inclusion criteria were as follows: (1) community-dwelling adults aged 65 years or above and speaking Chinese, (2) who had a Hong Kong ID card, and (3) who had not yet received SIV in the 2021–2022 flu season. Participants who were unable to communicate with interviewers were excluded. The target sample size was 400. We assumed that 45% of the participants intended to take up SIV, and 10–40% would complete the SIV in the reference group, which did not receive any facilitating condition. With a statistical power of 0.80 and an alpha value of 0.05, the smallest odds of 1.76 between the two groups with or without a facilitating condition could be detected (PASS 11.0, NCSS, LLC, Kaysville, UT, USA). In this study, 440 participants completed the survey, which was higher than our target sample size. As an illustration of the statistical power, the sample size could detect the smallest odds ratios of 1.72 based on the aforementioned scenarios.

### 2.3. Data Collection

We used simple random sampling, and inputted all the household telephone numbers (about 350,000) in Hong Kong into an Excel sheet. Telephone numbers were from the up-to-date telephone directories. Around 4000 telephone numbers were randomly selected by the random selection function of Excel. Via telephone interview, participants’ information was collected by trained interviewers during 6:00–10:00 p.m. and 2:00–9:00 p.m. on weekdays and Saturdays, respectively, to avoid under-sampling of working people. Households were considered as “non-valid” if no one answered any of five attempts of calling at different timeslots. The individual whose birthday was closest to the survey date was included if there was more than one individual aged ≥ 65 years in a household. Eligibility was screened, then interviewers briefed prospective eligible participants about the study details and procedure. Respondents’ anonymity was guaranteed, and participants had the right to quit without any consequences at any time. We recruited 440 participants after calling 3963 households; the remaining 37 households were not contacted. Among the 3963 households, 698 eligible older adults were identified, 258 of them refused to join the study, and 440 completed the telephone survey. Interviewers confirmed that participants fully understood the briefing and their willingness to participate in the study. Verbal instead of written informed consent was obtained to protect confidentiality. The same methods have been used in previous studies [35,36,37]. The survey took about 20 min to complete. No incentive was offered to participants. Ethics approval was obtained from the Survey and Behavioural Research Ethics Committee of the Chinese University of Hong Kong (SBRE-19-187).

### 2.4. Measures

#### 2.4.1. Design of the Questionnaire

A panel including researchers of public health, behavioural health, and vaccination behaviours developed the questionnaire. A pilot test was conducted among 10 older adults, who did not participate in the actual survey, to assess the questionnaire’s clarity and readability. All participants in the pilot test found the questionnaire easy to understand and believed that the length of the questionnaire was acceptable. Based on their feedback, the panel revised and finalised the questionnaire for the present study. The questionnaire in both English and Cantonese was shown in Material S1.

#### 2.4.2. Background Characteristics

Demographic characteristics (e.g., age, sex assigned at birth, education level, marital status), and lifestyle (i.e., smoking, and binge drinking in the past year) were collected. Participants also reported the presence of chronic diseases, COVID-19 history, and history of SIV and pneumococcal vaccination.

#### 2.4.3. Behavioural Intention to Receive SIV for the Incoming Flu Season

Participants were asked about the chance of receiving free SIV in the incoming flu season at the end of the interview (response categories ranged from 1 = very unlikely, 2 = unlikely, 3 = neutral, 4 = likely, to 5 = very likely). Participants who responded “likely” or “very likely” were defined as having a behavioural intention to take up SIV.

#### 2.4.4. COVID-19 Vaccination Uptake and Perceptions Related to COVID-19 and COVID-19 Vaccination

Participants reported the number of COVID-19 vaccination doses received by them. One item compared the infectivity of COVID-19 and seasonal influenza: “Is the infectivity of seasonal influenza virus lower or higher compared with COVID-19?” (response categories: 1 = lower than COVID-19, 2 = no different, 3 = higher than COVID-19, and 4 = unclear). Another item measured the perceived chance of co-infection of seasonal influenza and COVID-19: “If you do not receive seasonal influenza vaccination, how high is your chance of having co-infection of seasonal influenza and COVID-19 in the incoming flu season?” (response categories: 1 = very low, 2 = low, 3 = moderate, 4 = high, and 5 = very high). In addition, two items measured potential interactions between SIV and COVID-19 vaccination: “Seasonal influenza vaccination would negatively affect the effectiveness of COVID-19 vaccination” and “COVID-19 vaccination would negatively affect the effectiveness of seasonal influenza vaccination” (response categories: 1 = disagree, 2 = neutral, and 3 = agree).

#### 2.4.5. Knowledge and Perceptions Related to SIV

Three items were used to measure knowledge related to SIV. The knowledge score was calculated by adding the number of appropriate responses to each question (ranging from 0 to 3). We used the Health Belief Model (HBM) as the theoretical framework and adapted the validated scales to measure perceptions related to SIV [20,21]. These scales included the Perceived Benefit Scale (3 items), the Perceived Barrier Scale (4 items), the Cue to Action Scale (2 items), and the Perceived Self-efficacy Scale (2 items). Responses to these scales ranged from 1= disagree, 2 = neutral to 3 = agree. The Cronbach’s alpha of these scales ranged from 0.62 to 0.84, with the exception of the Perceived Barrier Scale (0.31). Therefore, we used four individual item responses instead of the score of the summative scale in the logistic regression analysis. In addition, two validated items measured perceived susceptibility and perceived severity of seasonal influenza: “If you do not receive seasonal influenza vaccination how high is your chance of having seasonal influenza in the incoming flu season?” and “If you do not receive seasonal influenza vaccination, how high is your chance of having severe illness due to seasonal influenza?” (response category: 1 = very low, 2 = low, 3 = moderate, 4 = high, and 5 = very high), respectively.

#### 2.4.6. Peer Influence Related to SIV

Two items measured perceived peer influence related to SIV. They were “Your family or friends had a history of seasonal influenza” and “In Hong Kong, how many people of your age would take up seasonal influenza vaccination for the incoming flu season?” (response categories: 1 = very few, 2 = few, 3 = some, 4 = many, and 5 = great many).

### 2.5. Statistical Analyses

We presented the frequency distribution of all studied variables and mean (standard deviation [SD]) of scale/item scores. Cronbach’s alpha for the scales was established by reliability tests. Univariate logistic models were used to estimate the associations of background variables with the dependent variable (i.e., behavioural intention to take up SIV); crude odds ratios (ORs) were obtained. After adjusting for background variables with *p* < 0.05 in the univariate analysis, the associations between independent variables of interest and the dependent variable were assessed by adjusted ORs (AORs). The 95% confidence intervals (CIs) for ORs and AORs were also presented. We performed statistical analyses using the R software (version 4.1.2, St Louis, MO, USA). *p* values below 0.05 (two-sided) were considered as statistically significant.

## 3. Results

### 3.1. Background Characteristics

About half of the participants were aged 65–69 years (49.8%) and had a secondary education level (47.5%). The majority of them were female (61.1%), and married or cohabiting with a partner (74.3%). Over 70% of participants had a household income of <HKD 20,000 (74.5%), were unemployed/retired/housewife (85.7%), and lived with others (81.6%). Few participants smoked (7.0%) or binge drank (2.3%) in the past year. Most participants reported at least one chronic disease (60.9%) and were without a COVID-19 history (98.2%). When the survey was conducted, 25.2% had taken up pneumococcal vaccination, and 34.5% had received three or more doses of SIV in the past three years (Table 1).

### 3.2. Descriptive Statistics of the Dependent Variable and Independent Variables of Interest

Over half of the participants (55.7%) were likely/very likely to take up SIV in the incoming flu season. Among the participants, 60.7% had received at least one dose of the COVID-19 vaccination. About half of the participants did not know the infectivity of COVID-19 versus seasonal influenza (40.7% reporting uncertainty and 7.7% perceiving no difference), and 37.5% perceived higher infectivity of COVID-19 than seasonal influenza. About 10.7% perceived a high/very high chance of having co-infection of seasonal influenza and COVID-19 without SIV, and 5.9% were concerned about the negative effect of SIV on COVID-19 vaccination effectiveness. Item responses and scale scores of knowledge, perceptions and peer influences related to SIV are shown in Table 2.

### 3.3. Factors Associated with a Behavioural Intention to Take up SIV

In univariate analyses, older age, being female, history of pneumococcal vaccination, and more doses of SIV received by the participants in the past three years were associated with a higher behavioural intention to take up SIV (*p* values from <0.001 to 0.03). However, compared to the unemployed/retired/housewife respondents, those with full-time/part-time employment were negatively associated with a behavioural intention to take up SIV (*p* = 0.003) (Table 3).

After adjustment for the above significant background characteristics, perceiving COVID-19 to have a higher infectivity than seasonal influenza (AOR: 1.93, 95% CI: 1.05, 3.56), and perceived higher risk of co-infection with COVID-19 and seasonal influenza (AOR: 3.07, 95% CI: 1.90, 4.94) were associated with a higher behavioural intention to take up SIV. Concerns that SIV would negatively affect the effectiveness of COVID-19 vaccination (AOR: 0.50, 95% CI: 0.30, 0.85) and COVID-19 vaccination would negatively affect the effectiveness of SIV (AOR: 0.50, 95% CI: 0.29, 0.86) were associated with a lower intention to receive SIV. In addition, history of COVID-19 vaccination was marginally associated with a behavioural intention to receive SIV (AOR: 1.65, 95% CI: 0.96, 2.84). Participants who perceived the consequences of seasonal influenza to be severe (AOR: 3.30, 95% CI: 2.02, 5.40), perceived higher benefits of SIV (AOR: 1.59, 95% CI: 1.33, 1.89), and received cues to action from significant others (AOR: 2.31, 95% CI: 1.69, 3.14) showed a higher intention to take up SIV. Concerns about severe side effects of SIV (AOR: 0.28, 95% CI: 0.16, 0.48), and the belief that one’s health conditions were not suitable for SIV (AOR: 0.56, 95% CI: 0.40, 0.80) were associated with a lower behavioural intention to take up SIV. In addition, perceiving that more people of their age in Hong Kong would take up SIV was associated with a higher behavioural intention (AOR: 2.08, 95% CI: 1.15, 3.76), while having a family member/friend with a history of seasonal influenza was associated with a lower intention to take up SIV (AOR: 0.59, 95% CI: 0.35, 0.98) (Table 4).

## 4. Discussion

This is one of the first studies investigating the associations of COVID-19 vaccination and perceptions related to COVID-19 with a behavioural intention to receive SIV among older adults in China during the COVID-19 pandemic. About half of the participants intended to receive SIV for the incoming flu season. The relatively low intention rate might explain the low and decreasing SIV uptake among local older adults during the COVID-19 pandemic (44.7% and 40.4% in the 2020–2021 and 2021–2022 seasons, respectively) [9]. In contrast to the findings in other places [27], the COVID-19 outbreak did not largely increase older adults’ motivation to receive SIV. The intention rate observed in our study was slightly higher than the time before COVID-19 (46.2% in 2006) [38]. Hong Kong started to resume normal life by relieving strict COVID-19 control measures in April 2022 [39]. There are concerns about seasonal outbreaks of COVID-19 and seasonal influenza [40]. Therefore, there is a strong need to improve SIV coverage among older adults.

In line with previous findings [19,41], participants with older age had a higher behavioural intention to receive SIV. They might perceive a stronger need to receive SIV due to poorer health conditions [42]. Being male was associated with a lower behavioural intention. As compared to males, females tend to be more concerned about their health status [43]. Future SIV programs should be aware of the gender difference in SIV acceptance and pay more attention to males. A history of SIV and pneumococcal vaccination was positively associated with a behavioural intention to receive SIV. Older adults with a history of these vaccinations might have a stronger motivation toward using vaccines to prevent infectious diseases [35]. A sizable proportion of older adults in Hong Kong have full-time/part-time employment [44]. As compared to those who were retired, older adults with employment had a lower intention to receive SIV. One possible reason might be lack of time due to conflict with working hours.

This study had numerous implications to strengthen the SIV promotion campaigns in Hong Kong. Our findings suggested that perceptions related to COVID-19 and COVID-19 vaccination were significant determinants of behavioural intention to receive SIV among older adults. In contrast to the findings in other places, COVID-19 vaccination uptake was not associated with a higher intention to receive SIV among older adults in Hong Kong. Such an insignificant association might be attributed to the concern about whether the COVID-19 vaccination and SIV would have a negative impact on each other, which was found to be barrier to receiving SIV in this study. Such a concern reflected older adults’ interpretation of the government’s recommendation of a 14-day interval between SIV and COVID-19 vaccination at the time of the survey [34]. Recent evidence shows that co-administration of the COVID-19 vaccination and SIV is safe and efficacious [45]. Such information should be disseminated to older adults. Moreover, the Hong Kong government started to promote and implement co-administration of SIV and COVID-19 vaccination in March 2023 [46], which may further reduce concerns among older adults. Only 10% of the participants perceived a high risk of co-infection with COVID-19 and seasonal influenza. Similar to the findings in other populations [29,30,47], perceived higher risk of co-infection with COVID-19 and seasonal influenza was associated with a higher behavioural intention to receive SIV. Health communication messages should enhance the perceived risk of such co-infection among older adults, and there is much room for improvement.

Our findings also showed that modifying perceptions related to SIV is still necessary when promoting SIV during the pandemic. Most participants did not perceive seasonal influenza as a serious health threat, as very few of them perceived a high risk of seasonal influenza or believed the consequences of seasonal influenza to be severe. Older adults who perceived higher susceptibility and severity of seasonal influenza had a higher behavioural intention. The fear appeal approach may be useful for promoting SIV among older adults [48]. The majority of the participants perceived some benefits of SIV. Future SIV promotional campaigns should strengthen these beliefs, as perceived benefits were associated with a higher intention. Information about the promising efficacies of SIV in protecting against seasonal influenza should be conveyed to older adults. In addition, health promotion should emphasise the effects of herd immunization, which would protect their family members who do not receive seasonal influenza. Belief in herd immunization was a facilitator to received pneumococcal vaccination among older adults in Hong Kong [36]. Barriers such as concerns about side effects and the belief that older adults are not suitable to receive SIV should be removed. Future health promotion should emphasise that SIV is very safe and involve positive experiences shared by vaccinated peers. Primary care physicians may play an important role in future SIV promotional campaigns, as they can perform assessments to assure whether an older adult is suitable to receive SIV. Their advice may serve as strong cue to action, which was significant facilitator of behavioural intention to receive SIV in this study. Furthermore, future programmes should also involve additional significant others of older adults such as family members and friends, as suggestions made by these significant others were also facilitators.

This study also observed significant influence from peers on the decision to receive SIV. The perception that more people of their age would receive SIV was associated with a higher behavioural intention to receive SIV. The Social Learning Theory suggests that observation of peers has a large influence on people’s attitudes and behaviours [49]. Peers’ experiences and information are perceived to be credible by older adults due to high rapport and trust [36]. Having a family member/friend with seasonal influenza history was associated with a lower behavioural intention to take up SIV. It is possible that these family members will share some information not supportive of SIV (e.g., seasonal influenza is not severe, and receiving the SIV cannot prevent seasonal influenza).

This study had several limitations. First, the study was conducted at the end of 2021 and early 2022 when strict control measures were applied in Hong Kong. Hong Kong lifted all strict control measures and resumed normal in March 2023. Second, selection bias was unavoidable due to non-response. Characteristics between participants and those refusing to participate could not be compared as we did not collect information from refusals. However, our response rate was comparable to previous studies with random telephone surveys on vaccination behaviours for older adults [35,36,37]. Third, recall bias could not be avoided as data were self-reported and verification was not feasible. Fourth, the study was cross-sectional and causal relationship could not be established. Last but not the least, generalization of the results to other cities and populations should be taken with caution.

## 5. Conclusions

Behavioural intention to take up SIV during the COVID-19 pandemic was relatively low among older adults in Hong Kong, China. Future programmes promoting SIV among older adults should address perceptions related to COVID-19 vaccination and SIV at the same time, as they were both significant determinants of behavioural intention to receive SIV in this group.

## Figures and Tables

**Table 1:** Participants’ background characteristics.

	*n*	%
**Socio-demographic characteristics**		
Age group, years		
65–69	219	49.8
70–74	147	33.4
≥75	74	16.8
Sex assigned at birth		
Male	171	38.9
Female	269	61.1
Highest education level obtained		
Primary or below	187	42.5
Secondary	209	47.5
Tertiary or above	44	10.0
Marital status		
Currently single	113	25.7
Married or cohabited with a partner	327	74.3
Household income, HKD (USD)/month		
<20,000 (2580)	328	74.5
≥20,000 (2580)	58	13.2
Refuse to disclose	54	12.2
Employment status		
Unemployed/retired/housewife	377	85.7
Full-time/part-time	63	14.3
Living alone		
No	359	81.6
Yes	81	18.4
Receiving Comprehensive Social Security Assistance ^a^		
No	408	92.7
Yes	32	7.3
**Lifestyles and health conditions**		
Smoking in the past year		
No	409	93.0
Yes	31	7.0
Binge drinking in the past year		
No	430	97.7
Yes	10	2.3
Presence of chronic conditions, Yes		
Hypertension	212	48.2
Other chronic cardiovascular disease	46	10.5
Chronic lung disease	8	1.8
Chronic liver disease	10	2.3
Chronic kidney disease	3	0.7
Diabetes mellitus	83	18.9
Any of above	268	60.9
History of COVID-19		
No	432	98.2
Yes	8	1.8
**Uptake of vaccinations**		
History of pneumococcal vaccination		
No	329	74.8
Yes	111	25.2
Number of seasonal influenza vaccination doses received by the participants in the past three years		
0	198	45.0
1	38	8.6
2	52	11.8
≥3	152	34.5

^a^: Comprehensive Social Security Assistance is a governmental financial support scheme providing a safety net for Hong Kong residents who cannot support themselves financially.

**Table 2:** Descriptive statistics of behavioural intention to receive seasonal influenza vaccination and independent variables of interest.

	*n*	%
Behavioural intention to receive seasonal influenza vaccination		
How high is your chance to receive a free seasonal influenza vaccination in the incoming flu season?		
Very unlikely, unlikely, or neutral	195	44.3
Likely or very likely	245	55.7
COVID-19 vaccination status		
Number of doses of COVID-19 vaccination received by the participants		
0	173	39.3
≥1	267	60.7
Perceptions related to COVID-19 and COVID-19 vaccination		
Which virus of COVID-19 and seasonal influenza has higher infectivity?		
Uncertain	179	40.7
COVID-19	165	37.5
No different	34	7.7
Seasonal influenza	62	14.1
If you do not receive seasonal influenza vaccination, how high is your chance of having seasonal influenza and COVID-19 co-infection in the incoming flu season, high or very high	47	10.7
Item score, mean (SD)	1.5	0.7
Seasonal influenza vaccination would reduce the effectiveness of COVID-19 vaccination, agree	26	5.9
Item score, mean (SD)	1.8	0.5
COVID-19 vaccination would reduce the effectiveness of seasonal influenza vaccination, agree	23	5.2
Item score, mean (SD)	1.8	0.5
Knowledge related to seasonal influenza vaccination, appropriate response		
Does the Hong Kong government recommend seasonal influenza vaccination to individuals aged 65 years or above?	429	97.5
Can all individuals aged 65 years or above receive free seasonal influenza vaccination at public hospitals/clinics?	428	97.3
Do you need to take up seasonal influenza vaccination every year?	425	96.6
Knowledge score, mean (SD)	2.9	0.4
Perceptions related to seasonal influenza vaccination		
Perceived susceptibility related to seasonal influenza, high/very high		
If you do not receive seasonal influenza vaccination how high is your chance of having seasonal influenza in the incoming flu season?	59	13.4
Item score, mean (SD)	1.5	0.7
Perceived severity of seasonal influenza, high or very high		
If you do not receive seasonal influenza vaccination, how high is your chance of having severe illness (e.g., bronchitis, pneumonia, brain lesions or death) due to seasonal influenza?	57	13.0
Item score, mean (SD)	1.5	0.7
Perceived benefit of seasonal influenza vaccination, agree		
Seasonal influenza vaccination is highly effective in protecting you from seasonal influenza	282	64.1
Seasonal influenza vaccination is highly effective in preventing severe consequences of seasonal influenza	302	68.6
Seasonal influenza vaccination is highly effective in protecting your family members from seasonal influenza	216	49.1
Perceived Benefit Scale, mean (SD)	7.5	1.8
Perceived barrier to receiving seasonal influenza vaccination, agree		
Seasonal influenza vaccination has severe side effects	31	7.0
Item score, mean (SD)	1.3	0.6
Seasonal influenza vaccination is too expensive for you	8	1.8
Item score, mean (SD)	1.1	0.4
It is inconvenient for you to receive seasonal influenza vaccination	13	3.0
Item score, mean (SD)	1.1	0.4
Your health conditions are not suitable for seasonal influenza vaccination	70	15.9
Item score, mean (SD)	1.5	0.8
Perceived Barrier Scale, mean (SD)	5.0	1.2
Cue to action related to seasonal influenza vaccination, agree		
Doctors would support you to receive seasonal influenza vaccination	299	68.0
Your family or friends would support you to receive seasonal influenza vaccination	213	48.4
Cue to Action Scale, mean (SD)	5.0	1.0
Perceived self-efficacy related to receiving seasonal influenza vaccination, agree		
You are confident to receive seasonal influenza vaccination if you want to	422	95.9
Taking up seasonal influenza vaccination is easy for you	416	94.5
Perceived Self-efficacy Scale, mean (SD)	5.9	0.6
Peer influence related to seasonal influenza vaccination		
Your family or friends had history of seasonal influenza		
No	219	40.8
Yes	221	50.2
In Hong Kong, how many people of your age would receive seasonal influenza vaccination for the incoming flu season?		
Very few/few/some	289	61.1
Many/great many	151	38.9

SD: standard deviation.

**Table 3:** Background characteristics associated with behavioural intention to receive seasonal influenza vaccination.

	Behavioural Intention to Take up SIV, % Yes	OR (95% CI)	*p* Values
**Socio-demographic characteristics**			
Age group, years			
65–69	50.2	1.00	
70–74	61.9	1.61 (1.05, 2.46)	0.03
≥75	59.5	1.45 (0.85, 2.48)	0.17
Sex assigned at birth			
Male	49.1	1.00	
Female	59.9	1.54 (1.05, 2.27)	0.03
Highest education level obtained			
Primary or below	54.0	1.00	
Secondary	56.0	1.08 (0.73, 1.61)	0.69
Tertiary or above	61.4	1.35 (0.69, 2.65)	0.38
Marital status			
Currently single	58.4	1.00	
Married or cohabited with a partner	54.7	0.86 (0.56, 1.33)	0.50
Household income, HKD (USD)/month			
<20,000 (2580)	54.3	1.00	
≥20,000 (2580)	62.1	1.38 (0.78, 2.45)	0.27
Refuse to disclose	57.4	1.14 (0.63, 2.03)	0.67
Employment status			
Unemployed/retired/housewife	58.6	1.00	
Full-time/part-time	38.1	0.43 (0.25, 0.75)	0.003
Living alone			
No	54.0	1.00	
Yes	63.0	1.45 (0.88, 2.38)	0.15
Receiving Comprehensive Social Security Assistance ^a^			
No	55.9	1.00	
Yes	53.1	0.89 (0.43, 1.84)	0.76
**Lifestyles and health conditions**			
Smoking in the past year			
No	56.5	1.00	
Yes	45.2	0.63 (0.30, 1.32)	0.22
Binge drinking in the past year			
No	55.6	1.00	
Yes	60.0	1.20 (0.33, 4.31)	0.78
Presence of any chronic conditions			
No	51.2	1.00	
Yes	58.6	1.35 (0.92, 1.98)	0.13
History of COVID-19			
No	56.3	1.00	
Yes	25.0	0.36 (0.05, 1.30)	0.10
**Uptake of vaccinations**			
History of pneumococcal vaccination			
No	44.4	1.00	
Yes	89.2	10.34 (5.47, 19.56)	<0.001
Number of SIV doses received by the participants in the past three years			
0	20.7	1.00	
1	57.9	5.27 (2.54, 10.92)	<0.001
2	78.8	14.27 (6.75, 30.19)	<0.001
≥3	92.8	49.08 (24.29, 99.16)	<0.001

OR: odds ratio, CI: confidence interval, SIV: seasonal influenza vaccination. ^a^: Comprehensive Social Security Assistance is a governmental financial support scheme providing a safety net for Hong Kong residents who cannot support themselves financially.

**Table 4:** Factors associated with behavioural intention to receive seasonal influenza vaccination.

	OR (95% CI)	*p* Values	AOR (95% CI)	*p* Values
COVID-19 vaccination status				
Number of doses of COVID-19 vaccination received by the participants				
0	1.00		1.00	
≥1	1.61 (1.09, 2.37)	0.02	1.65 (0.96, 2.84)	0.07
Perceptions related to COVID-19 and COVID-19 vaccination				
Which virus of COVID-19 and seasonal influenza has higher infectivity?				
Uncertain	1.00		1.00	
COVID-19	1.76 (1.14, 2.70)	0.01	1.93 (1.05, 3.56)	0.04
No different	1.34 (0.64, 2.80)	0.44	1.71 (0.64, 4.59)	0.29
Seasonal influenza	1.46 (0.82, 2.62)	0.20	2.12 (0.95, 4.73)	0.07
If you do not receive seasonal influenza vaccination, how high is your chance of having seasonal influenza and COVID-19 co-infection in the incoming flu season?	3.51 (2.43, 5.05)	<0.001	3.07 (1.90, 4.94)	<0.001
Seasonal influenza vaccination would reduce the effectiveness of COVID-19 vaccination	0.31 (0.21, 0.47)	<0.001	0.50 (0.30, 0.85)	0.01
COVID-19 vaccination would reduce the effectiveness of seasonal influenza vaccination	0.31 (0.20, 0.46)	<0.001	0.50 (0.29, 0.86)	0.01
Knowledge related to seasonal influenza vaccination				
Knowledge score	1.07 (0.67, 1.69)	0.79	0.67 (0.38, 1.19)	0.17
Perceptions related to seasonal influenza vaccination				
Perceived susceptibility ^a^	4.57 (3.11, 6.72)	<0.001	3.59 (2.18, 5.89)	<0.001
Perceived severity ^a^	3.61 (2.51, 5.19)	<0.001	3.30 (2.02, 5.40)	<0.001
Perceived Benefit Scale	1.86 (1.62, 2.14)	<0.001	1.59 (1.33, 1.89)	<0.001
Perceived barrier ^a^				
Seasonal influenza vaccination has severe side effects	0.15 (0.10, 0.25)	<0.001	0.28 (0.16, 0.48)	<0.001
Seasonal influenza vaccination is too expensive for you	0.99 (0.59, 1.66)	0.96	0.94 (0.45, 1.96)	0.88
It is inconvenient for you to receive seasonal influenza vaccination	1.61 (0.89, 2.91)	0.12	2.05 (0.94, 4.49)	0.07
Your health conditions are not suitable for seasonal influenza vaccination	0.52 (0.40, 0.68)	<0.001	0.56 (0.40, 0.80)	0.001
Cue to Action Scale	3.62 (2.78, 4.72)	<0.001	2.31 (1.69, 3.14)	<0.001
Perceived Self-efficacy Scale	1.64 (1.14, 2.38)	0.01	1.42 (0.88, 2.28)	0.15
Peer influence related to seasonal influenza vaccination				
Your family or friends had history of seasonal influenza				
No	1.00		1.00	
Yes	0.51 (0.35, 0.75)	<0.001	0.59 (0.35, 0.98)	0.04
In Hong Kong, how many people of your age would receive seasonal influenza vaccination for the incoming flu season?				
Very few/few/some	1.00		1.00	
Many/great many	3.61 (2.27, 5.73)	<0.001	2.08 (1.15, 3.76)	0.02

OR: odds ratio, CI: confidence interval, AOR: adjusted odds ratio, adjusted for significant background characteristics in Table 3. ^a^: Item scores were used for data analyses.

## Data Availability

The data presented in this study are available from the corresponding author upon request. The data are not publicly available as they contain personal behaviours.

## Supplementary Materials

The following supporting information can be downloaded at: https://www.mdpi.com/article/10.3390/vaccines11071213/s1, Material S1: Questionnaires in both English and Cantonese.
