# Supplementary material for: Associations between COVID-19 Vaccination and Behavioural Intention to Receive Seasonal Influenza Vaccination among Chinese Older Adults: A Population-Based Random Telephone Survey
Source: Vaccines (Basel). 2023 Jul 6;11(7):1213. doi: 10.3390/vaccines11071213 (PMC10385482; doi:10.3390/vaccines11071213)
Supplement: Supplementary file 1 [file vaccines-11-01213-s001.zip › vaccines-2458186-supplementary.pdf]

Material S1: Questionnaires in both English and Cantonese.

Part 1

Q1a 請問係過去三年中(由 2018 年開始計)，你打過幾次流感針?

How many doses of seasonal influenza vaccination have you received in the past three years (since 2018)?

☐1 0 ☐2 1 ☐3 2 ☐4 3

關於流感針的看法

Q2 如果你唔打流感針，你認為出現以下情況嘅機會有幾大<br>If you do not receive seasonal influenza vaccination, how high is your chance of having the following condition?	非常低<br>Very low	比較低<br>Low	中等<br>Moderate	比較高<br>High	非常高<br>Very high
a) 在下一個流感季患上流感嘅機會<br>Your chance of having seasonal influenza in the incoming flu season?	1	2	3	4	5
b) 因患上流感而出現嚴重病情（例如支氣管炎、肺炎、腦病變或者死亡）嘅機會<br>Your chance of having severe illness (e.g., bronchitis, pneumonia, brain lesions or death) due to seasonal influenza	1	2	3	4	5
c) 下一個流感季同時患上流感同新冠肺炎嘅機會<br>Your chance of having co-infection of seasonal influenza and COVID-19 in the incoming flu season	1	2	3	4	5

Q3 同新冠肺炎病毒相比，流感病毒嘅傳染性更高定係更低?

Which virus of COVID-19 and seasonal influenza has higher infectivity?

☐1 新冠肺炎病毒 (COVID-19) ☐2 冇分別 (No different)

☐3 流感病毒 (seasonal influenza) ☐4 唔清楚 (Uncertain)

Q4 關於流感針

a) 香港政府有冇推薦 65 歲以上嘅長者打流感針?

Does the Hong Kong government recommend seasonal influenza vaccination to individuals aged 65 years or above?

☐1 有 (Yes) ☐2 冇 (No) ☐3 唔清楚 (Uncertain)

b) 65 歲以上嘅長者是否都可以在公立醫院/診所免費打流感針?

Can all individuals aged 65 years or above receive free seasonal influenza vaccination at public hospitals/clinics?

☐1 可以 (Yes) ☐2 不可以(No) ☐3 唔清楚 (Uncertain)

c) 流感針是否需要每年都打?

Do you need to take up seasonal influenza vaccination every year?

☐1 有 (Yes) ☐2 冇 (No) ☐3 唔清楚 (Uncertain)

Q5 我地想知道你對流感針嘅一啲看法 (Attitudes toward seasonal influenza vaccination)

	同意<br>Agree	不同意<br>Disagree	中立<br>Neutral
a) 打流感針可以有效預防你患上流感<br>Seasonal influenza vaccination is highly effective in protecting you from seasonal influenza	1	2	3
b) 打流感針可以有效預防你因感染流感而出現嚴重病情	1	2	3

Seasonal influenza vaccination is highly effective in preventing severe consequences of seasonal influenza			
c) 你打流感針有助於保護你嘅屋企人唔患上流感<br>Seasonal influenza vaccination is highly effective in protecting your family members from seasonal influenza	1	2	3
d) 流感針有比較嚴重嘅副作用<br>Seasonal influenza vaccination has severe side effects	1	2	3
e) 你覺得打流感針嘅收費比較貴<br>Seasonal influenza vaccination is too expensive for you	1	2	3
f) 打流感針嘅時間同地點對於你嚟講唔方便<br>It is inconvenient for you to receive seasonal influenza vaccination	1	2	3
g) 你覺得你嘅身體狀況唔適合打流感針<br>Your health conditions are not suitable for seasonal influenza vaccination	1	2	3
h) 流感針會降低新冠肺炎疫苗嘅效果<br>Seasonal influenza vaccination would negatively affect the effectiveness of COVID-19 vaccination	1	2	3
i) 新冠肺炎疫苗會降低流感針嘅效果<br>COVID-19 vaccination would negatively affect the effectiveness of seasonal influenza vaccination	1	2	3
j) 醫護人員會支持你打流感針<br>Doctors would support you to receive seasonal influenza vaccination	1	2	3
k) 你嘅屋企人或朋友會支持你打流感針<br>Your family or friends would support you to receive seasonal influenza vaccination	1	2	3
m) 你若果想打流感針，你有好大信心可以做到<br>You are confident to receive seasonal influenza vaccination if you want to	1	2	3
n) 打流感針對你嚟講係件容易嘅事<br>Taking up seasonal influenza vaccination is easy for you	1	2	3

Q6 係香港，你認為同你年紀相近嘅長者，有幾多會係下一個流感季打流感針？

In Hong Kong, how many people of your age would receive seasonal influenza vaccination for the incoming flu season?

☐1 非常少(Very few) ☐2 比較少(Few) ☐3 中等(Some)
☐4 比較多(Many) ☐5 非常多(Great many)

Q7 如果免费，請問你下一個流感季內打流感針嘅機會有幾高？

How high is your chance to receive free seasonal influenza vaccination in the incoming flu season?

☐1 可能性非常低 (very unlikely) ☐2 可能性比較低 (unlikely) ☐3 中立 (neutral)
☐4 可能性比較高 (likely) ☐5 可能性非常高 (very likely)

Part 2 History of other vaccination

Q8 請問你有冇打過肺炎球菌疫苗(23 價及/或 13 價)?

Have you ever received pneumococcal vaccination?

☐1 冇/唔記得 (No/uncertain) ☐2 打過 1 針 (received 1 dose) ☐3 打過 2 針 (received two doses)

Q9 請問你有冇打過新冠肺炎疫苗？

Have you ever received COVID-19 vaccination?

☐1 冇/唔記得 (No/uncertain) ☐2 打過 1 針 (received 1 dose) ☐3 打過 2 針 (received two doses)

Part 3 基本情況

Background characteristics

Q10 性別 (Gender) ☐1 男 (male) ☐2 女 (female)

Q11 年齡 (age) ________歲 (years)

Q12 教育程度 (Education level)

☐1 小學以下 (below primary school) ☐2 小一至小六 (primary school)
☐3 中一至中三 (junior high school) ☐4 中四至中五或者預科 (senior high school)
☐5 專上或大學 (college or undergraduate) ☐6 大學以上 (postgraduate)
☐7 其他，請註明 (Others, please be specific)____________

Q13 你而家嘅婚姻狀況係？(Relationship status)

☐1 未婚 (single) ☐2 已婚 (married) ☐3 分居/離婚 (separated/divorced)
☐4 喪偶 (widowed) ☐5 同居 (cohabited with a partner)
☐6 其他，請註明 (Others, please be specific)____________________

Q14 你而家嘅工作情況係？(Current employment status)

☐1 全職 (full-time) ☐2 兼職 (part-time) ☐3 退休 (retired)
☐4 待業/失業 (unemployed) ☐5 家庭主婦 (housewife)
☐6 其他，請註明 (Others, please be specific)____________________

Q15 你而家同邊個一齊住？（可多選）

Are you living with the following persons? (You can choose more than one response)

☐1 仔女 (children/grandchildren) ☐2 配偶 (spouse)
☐3 工人（菲佣）(domestic helpers) ☐4 其他人，請註明 (others, please be specific)_____________
☐5 獨居 (living alone)

Q16 你每月平均家庭收入係邊個範圍？(Monthly household income, in Hong Kong dollars)

☐1 20,000 以下 ☐2 20,000-39,999 ☐3 40,000-59,999 ☐4 60,000-79,999
☐5 80,000 或以上 (80,000 or above)
☐6 冇固定收入 (no stable income) ☐7 拒絕透露 (refuse to disclose)

Q17 請問你有冇領取綜援？

Are you receiving Comprehensive Social Security Assistance?

☐1 有 (Yes) ☐2 冇 (No)

Q18 我地想了解一下你目前嘅健康狀況 (Did you have the following health conditions?)

	有<br>Yes	冇<br>No	唔清楚<br>Uncertain
a) 高血壓 (hypertension)	1	2	3
b) 其他長期心血管疾病（例如心臟病、腦血管病等）(other chronic cardiovascular diseases)	1	2	3
c) 慢性肺病（例如慢性阻塞性肺病、肺癌等）(chronic lung diseases)	1	2	3
d) 慢性肝病（例如慢性肝炎、肝硬化、肝癌等）(chronic liver diseases)	1	2	3

	有<br>Yes	冇<br>No	唔清楚<br>Uncertain
e) 慢性腎病（例如慢性腎炎、慢性腎功能不全/腎衰竭、腎癌等）(chronic kidney diseases)	1	2	3
f) 糖尿病 (diabetes mellitus)	1	2	3
g) 曾確診患上新冠肺炎 (history of COVID-19)	1	2	3

Q19 據你所知，你嘅屋企人或朋友之前有冇患過流感？

Did your family or friends have a history of seasonal influenza?

☐1 有 (Yes) ☐2 冇 (No) ☐3 唔清楚 (Uncertain)

Q20 過去一年中，你平均每日食幾多支煙？

How many cigarettes did you smoke per day in the past year?

☐1 冇吸煙 (did not smoke) ☐2 唔夠 1 支 (less than one) ☐3 1-10 支 (1-10)

☐4 11-19 支 (11-19) ☐5 20-39 支 (20-39) ☐6 40 支或以上 (40 or more)

Q21 過去一年中，你有冇係一個場合內飲超過 5 罐啤酒，或者 5 細杯（茶杯）烈酒（高度數白酒或者洋酒），或者 5 杯紅/白葡萄酒？

Did you have at least 5 cans of beer/5 glasses of table wine/5 peg of spirits on one occasion in the past year?

☐1 有 (Yes) ☐2 冇 (No)
